# Complete Whole-Genome Sequence of Haemophilus haemolyticus NCTC 10839

**DOI:** 10.1128/MRA.00232-19

**Published:** 2019-06-20

**Authors:** Mohammed-Abbas Fazal, Sarah Alexander, Nicholas E. Grayson, Ana Deheer-Graham, Karen Oliver, Nancy Holroyd, Julian Parkhill, Julie E. Russell

**Affiliations:** a Culture Collections, Public Health England, London, United Kingdom; b Wellcome Trust Sanger Institute, Wellcome Trust Genome Campus, Hinxton, United Kingdom; University of Maryland School of Medicine

## Abstract

Haemophilus haemolyticus is a Gram-negative bacterium that is a commensal of the respiratory tract in humans. Here, we report the complete genome sequence available for Haemophilus haemolyticus strain NCTC 10839, which was originally isolated from the nasopharynx of a child.

## ANNOUNCEMENT

Haemophilus haemolyticus is a Gram-negative rod-shaped bacterium that colonizes the respiratory tract and is generally considered to be a commensal organism that is rarely involved in causing human disease (1). However, reports of invasive disease caused by H. haemolyticus, including endocarditis, have been reported (2, 3). Difficulties in distinguishing between the human pathogen Haemophilus influenzae and H. haemolyticus may have resulted in the underreporting of cases associated with disease due to misidentification as nontypeable H. influenzae (1, 4–6).

Currently, there are 45 genome sequences available for H. haemolyticus, 41 of which are draft genome sequences and 3 of which are complete genome sequences. Here, we report the complete whole-genome sequence from a strain of H. haemolyticus [NCTC 10839; strain A67(A)] which has been deposited within a culture collection and is therefore publicly available to the scientific community. This strain was deposited in the National Collection of Type Cultures in 1972 and was originally isolated from the nasopharynx of a child in Aarhus, Denmark.

Strain NCTC 10839 was recovered from a lyophilized culture in a glass ampule, placed onto chocolate blood agar, and incubated at 37°C for 24 hours. Genomic DNA was extracted from the resulting culture using the MasterPure DNA kit (catalog number MC85200; Epicentre) and underwent quality control for high-molecular-weight DNA (>60 kbp) with the Agilent 2200 TapeStation system and for high yield with the Quant-iT double-stranded DNA (dsDNA) broad-range (BR) assay kit (minimum, 3 μg DNA). Sequencing was performed on the Pacific Biosciences (PacBio) RS II platform. A 10- to 20-kb library was prepared and sequenced using C4-P6 chemistry on single-molecule real-time (SMRT) cells, with a 180-min collection protocol, on the PacBio RS II platform. Sequence reads were assembled using the Hierarchical Genome Assembly Process (HGAP) v3 (7) of the SMRT Analysis software v2.3.0. The fold coverage to target when picking the minimum fragment length for assembly was set to 30, and the approximate genome size was set to 3 Mbp. The assembly was circularized using Circlator v1.1.3 (8). Finally, the circularized assembly was polished using the PacBio RS_Resequencing protocol and Quiver v1 of the SMRT Analysis software v2.3.0. Automated annotation was performed using Prokka v1.5 (9) and a genus-specific database from RefSeq (10). The chromosome of NCTC 10839 was assembled as a single contig of 1,934,644 bp, with a GC content of 38.42% and an *N*_50_ value of 197,298 bp. The average read coverage for the assembly was 268×. No plasmid DNA was identified in the assembly. There were 1,756 protein-coding DNA sequence (CDS) genes, 60 tRNA genes, and 19 rRNA genes. No antibiotic resistance genes were identified by ResFinder v2.1 (11).

### Data availability.

The complete genome sequence has been deposited in the National Center for Biotechnology Information database under BioSample accession number SAMEA3905387 and BioProject number PRJEB6403. The Sequence Read Archive accession number is ERS1092521.
